# *Preventing Chronic Disease:* Recognizing Accomplishments in 2016 and Tracking Progress of Priorities in 2017

**DOI:** 10.5888/pcd14.170241

**Published:** 2017-06-08

**Authors:** Leonard Jack

**Figure:**
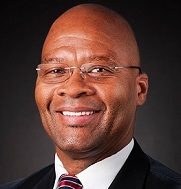
Leonard Jack, Jr, PhD, MSc, Editor in Chief

*Preventing Chronic Disease* (*PCD*) recently released its *2016 Year in Review*, which highlights several important accomplishments. Last year was a year of growth and change for the journal, both in editorial quality and technological advances. We welcomed several new associate editors, expanding our team of elite researchers and practitioners committed to advancing the science and practice of public health. Their expertise has brought a wealth of knowledge to the journal and will enhance our visibility and influence.

Again in 2016 the number of manuscripts submitted for consideration increased, demonstrating that *PCD* continues to attract top-notch research and practice submissions. Part of that increase may have resulted from our improved outreach — tailoring content to our audiences and expanding to new audiences. Last year, *PCD* launched its own Twitter handle to increase the journal’s social media reach and complement its Facebook posts. Our articles are referenced by an ever-increasing media base that includes prestigious news sources such as *Time*, *PBS NewsHour*, WebMD, and *ABC News*.

*PCD* continues to excel in technical innovation. In 2016, we redesigned our home page to make it more visually appealing and engaging to our visitors, and we added rotating graphics paired with the latest announcements. The journal also began partnering with Altmetric to track media and social media mentions of *PCD* articles. Altmetric data are nontraditional metrics proposed as an alternative to traditional citation-based metrics such as the impact factor. Altmetric data will help us to better measure the journal’s impact in traditional and social media and give us a clearer picture of our readers’ interests. Look for the colorful Altmetric badge on our article pages. For more information on other accomplishments last year, take a look at *PCD*’s *2016 Year in Review*: www.cdc.gov/PCD/about_the_journal/index.htm.

In my first column, published in February, I outlined what I envisioned as *PCD*’s Top 10 Priorities in defining the journal’s future direction. We have already made impressive progress on those priorities. We increased the number of associate editors from 7 editors in 2016 to 14 editors in 2017, and we are working on adding more. This diverse team of professionals has helped us address the priority of adopting a multilayered approach to reviewing and approving manuscripts, which allows us to better identify and streamline the acceptance of high-quality manuscripts. *PCD* also launched a new article type in April, Implementation Evaluation. Articles in this new peer-reviewed category will provide insight to program planners, policy makers, evaluators, researchers, and diverse stakeholders on factors that affect the ability of public health practitioners to package and disseminate effective interventions that have been implemented and evaluated in real-world settings (eg, clinics, hospitals, schools, communities, worksites). Implementation Evaluation addresses one of *PCD*’s Top 10 Priorities by embracing evaluation methods and approaches to assessing the complex mix of individual, population, organizational, economic, and environmental factors contributing to the success of interventions. Authors interested in submitting an Implementation Evaluation article should review the submission guidelines: www.cdc.gov/PCD/for_authors/types_of_articles.htm.

*PCD* has remained faithful to its priority to mentor future public health researchers and practitioners by sponsoring its annual Student Research Paper Contest. After extending the contest deadline from January to March, *PCD* received 72 manuscripts, the highest number of submissions since this contest began in 2013. For the first time, *PCD* will decide winners in categories based on level of education: doctoral, master’s, undergraduate, and high school. Entries in this year’s contest will undergo a rigorous and layered peer review process, and we appreciate the *PCD* editorial board members who agreed to serve on reviewing committees. We also thank the many students and faculty mentors for their interest and participation in this year’s contest. Look for our contest winners later this year.

To address the priority of ensuring scientific integrity and quality, *PCD* finalized its plans to host its first-ever external review, inviting 7 nationally respected scholars and practitioners to examine the journal’s mission, processes, scope, reach, and impact in advancing the science and practice of public health. *PCD* must continue to make improvements to better serve its readership; the *PCD* external review will provide the journal’s leadership with recommendations to shape *PCD*’s focus, function, and direction. In a future column, I intend to share the external review’s major findings and how they will be used to refine the journal’s future direction. We are pleased with progress to date on our priorities, and we look forward to making progress on all 10 priorities by the end of the year.

I end this Editor in Chief’s Column by sharing good news. The US Department of Health and Human Services was named by Reuters as the number-one publicly funded institution in “doing the most to advance science and technology.” As part of the department’s family of products at the Centers for Disease Control and Prevention, *PCD* shares in that honor. The Reuters rankings rely on data compiled by Clarivate Analytics and several research platforms, including inCites, Web of Science, Derwent Innovations Index, Derwent World Patents Index, and Patents Citation Index. The data identified more than 600 global organizations that published the most articles in academic journals. Candidates were evaluated by the number of articles published by researchers in academic journals, how often those articles were cited by patents, and how many articles featured a coauthor from industry. We are gratified to know that *PCD* continues to be a valuable resource to many audiences. This recognition is a reflection of the commitment to scientific integrity and quality by *PCD* authors, associate editors, editorial board members, peer reviewers, and staff members. The honor is one that should be celebrated by everyone. For more information on the rankings, please visit http://stateofinnovation.com/which-government-agencies-are-leading-the-world-in-innovation?utm_campaign=15244%20-%20Reuters%20Top%2025%20Global%20Govt%20Ranking_SSR_2017_Ranked%20-%2032901&utm_medium=email&utm_source=Eloqua.

